# Are FDA-Approved Sunscreen Components Effective in Preventing Solar UV-Induced Skin Cancer?

**DOI:** 10.3390/cells9071674

**Published:** 2020-07-11

**Authors:** Ann M. Bode, Eunmiri Roh

**Affiliations:** The Hormel Institute, University of Minnesota, 801 16th Avenue NE, Austin, MN 55912, USA; bodex008@umn.edu

**Keywords:** sunscreen, prevention, cutaneous squamous cell carcinoma, skin cancer, solar ultraviolet, skin carcinogenesis

## Abstract

Solar ultraviolet (SUV) exposure is a major risk factor in the etiology of cutaneous squamous cell carcinoma (cSCC). People commonly use sunscreens to prevent SUV-induced skin damage and cancer. Nonetheless, the prevalence of cSCC continues to increase every year, suggesting that commercially available sunscreens might not be used appropriately or are not completely effective. In the current study, a solar simulated light (SSL)-induced cSCC mouse model was used to investigate the efficacy of eight commonly used FDA-approved sunscreen components against skin carcinogenesis. First, we tested FDA-approved sunscreen components for their ability to block UVA or UVB irradiation by using VITRO-SKIN (a model that mimics human skin properties), and then the efficacy of FDA-approved sunscreen components was investigated in an SSL-induced cSCC mouse model. Our results identified which FDA-approved sunscreen components or combinations are effective in preventing cSCC development. Not surprisingly, the results indicated that sunscreen combinations that block both UVA and UVB significantly suppressed the formation of cutaneous papillomas and cSCC development and decreased the activation of oncoproteins and the expression of COX-2, keratin 17, and EGFR in SSL-exposed SKH-1 (Crl:SKH1-*Hr^hr^*) hairless mouse skin. Notably, several sunscreen components that were individually purported to block both UVA and UVB were ineffective alone. At least one component had toxic effects that led to a high mortality rate in mice exposed to SSL. Our findings provide new insights into the development of the best sunscreen to prevent chronic SUV-induced cSCC development.

## 1. Introduction

Developing effective prevention strategies against cutaneous squamous cell carcinoma (cSCC) non-melanoma skin cancer (NMSC) is extremely important because many people receive extensive exposure to sunlight early in life and develop skin cancers later in life even without further exposure to sunlight [1]. Because of the increasing incidence of NMSC, the effectiveness of sunscreens in preventing skin damage and cSCC is debatable.

Solar ultraviolet (SUV) radiation is an environmental carcinogen that can result in skin aging, inflammation, and skin cancer development, which is the most common malignancy worldwide in Caucasian populations [2,3]. Approximately 1/3 of all new cancers are skin cancers, and in particular the incidence of cSCC NMSC increases every year, with an estimated annual incidence of 700,000 in the USA [4,5]. As incidence rates for NMSC continue to rise, a substantial impact on morbidity and health care costs occurs and accounts for a significant financial burden in skin cancer treatment [6]. Basal cell carcinomas (BCCs) rarely metastasize, whereas cSCCs can metastasize to regional lymph nodes or other distant organs, resulting in death. The number of cSCC-related deaths is estimated to be between 3932 to 8791 annually and the upper limit approaches the total annual melanoma-related deaths [7,8]. Reducing the incidence of possibly dangerous cSCCs would not only reduce their potentially severe morbidity and mortality, but also reduce the multibillion-dollar costs associated with surgical and medical treatments required for NMSC. Actinic keratosis (AK) is believed to act as a precursor to cSCC. AKs are premalignant skin lesions induced by SUV and characterized by the proliferation of atypical keratinocytes confined to the epidermis [9,10,11,12]. Regardless of the type of treatment chosen, individuals with an increased risk of developing AKs should be advised to apply a protective sunscreen as preventive skin care.

Even though people widely use sunscreens to protect the skin against solar UV irradiation, the ability of sunscreens to prevent cSCC still remains controversial. Furthermore, people are using moisturizing lotions topically, but most of these lotions have not been tested for carcinogenic activity during the course of SUV radiation. Several commercially available moisturizing lotions reportedly increased the rate of formation and the number of tumors when applied topically to the skin of UVB-pretreated high-risk mice [13]. Thus, we made a non-toxic moisturizing lotion formulation and tested its safety in an in vivo mouse skin model (Patent International Application Number: PCT/US2018/036720; title: skin care formulations and skin cancer treatment). Furthermore, the prevalence of cSCC continues to increase annually, suggesting that commercially available sunscreens might not be used appropriately or are not completely effective. Thus, here we investigated the efficacy of the most commonly used Food and Drug Administration (FDA)-approved sunscreen components in an in vitro human skin model and in an in vivo SSL-induced cSCC mouse model.

The current FDA-approved list of sunscreen ingredients contains 16 components that can be included in sunscreens. However, only 8 of the 16 are regularly used in US-produced sunscreens, and only 2 of those offer full UVA protection. The eight most commonly used sunscreen components include octocrylene, oxybenzone [14], avobenzone [15,16], octinoxate, octisalate [17], homosalate [18], titanium dioxide [19], and zinc oxide [20]. The absorbance wavelengths of each FDA-approved sunscreen component and maximum peaks of UV absorption are shown in Supplementary Figure S1.

In the present study, we formulated a moisturizing lotion that neither prevents nor causes skin cancer (Patent International Application Number: PCT/US2018/036720; title: skin care formulations and skin cancer treatment). We used this base lotion as a vehicle to test the effectiveness of individual or combinations of FDA-approved sunscreen components [21,22] in protecting against SSL-induced damage and cSCC development.

## 2. Materials and Methods

### 2.1. Antibodies and Chemicals

**Figure 1:**
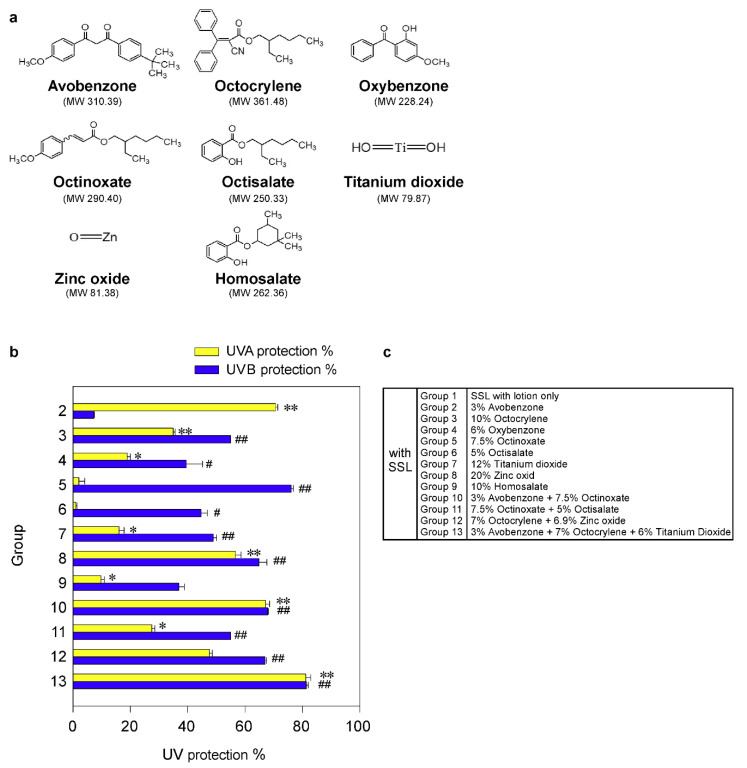
Effectiveness of FDA-approved sunscreen components in vitro. (**a**) Chemical structures of 8 FDA-approved sunscreen components tested herein. (**b**) The percentage of UV protection of sunscreens against UVA or UVB rays. The asterisks (*^,^ **) indicate a significant (*p* < 0.05 or *p* < 0.01, respectively) difference compared to the only solar simulated light (SSL)-exposed control group with lotion (group 1) for UVA protection. The asterisks (^#, ##^) indicate a significant (*p* < 0.05 or *p* < 0.01, respectively) difference compared to the only SSL-exposed control group with lotion (group 1) for UVB protection. (**c**) Group information is presented.

The phospho-PRPK (Ser250) antibody was generated by BioSynthesis, Inc. (Lewisville, TX, USA), and the phospho-TOPK (Thr9) antibody (Catalog no. 12233) was purchased from Signalway Antibody LLC (College Park, MD, USA). The TOPK (Catalog no. CST-4942), phospho-EGFR (Tyr1068, Catalog no. CST-3777), and keratin 17 (Catalog no. CST-4543) antibodies were purchased from Cell Signaling Technologies (Danver, MA, USA). The PRPK antibody (Catalog no. sc-100350) was purchased from Santa Cruz Biotechnologies (Santa Cruz, CA, USA) and the COX-2 (Catalog no. 160106) antibody was obtained from Cayman (Ann Arbor, MI, USA). The Ki-67 antibody (Catalog no. RM-9106) was from Thermo Fisher Scientific (Waltham, MA, USA). Avobenzone (CAS no. 70356-09-1), titanium dioxide (CAS no. 13463-67-7), and homosalate (CAS no. 118-56-9) were purchased from Thermo Fisher Scientific. Octocrylene (CAS no. 6197-30-4), oxybenzone (CAS no. 131-57-7), octinoxate (CAS no. 5466-77-3), octisalate (CAS no. 118-60-5), and zinc oxide (CAS no. 1314-13-2) were obtained from Sigma-Aldrich (St. Louis, MO, USA). The chemical structures of each sunscreen component are shown in Figure 1a. All the other chemicals, including lotion ingredients, were purchased from Sigma-Aldrich and Thermo Fisher Scientific.

### 2.2. Solar Simulated Light (SSL) Source for Experiments

The SSL source comprised UVA-340 lamps purchased from Q-Lab Corporation (Cleveland, OH, USA). This SSL system of irradiation mimics natural sunlight that emits both UVA and UVB wavelengths and provides the best possible simulation of sunlight. UVA-340 lamps provide both UVA and UVB. The percentage of UVA and UVB emitted from the UVA-340 lamps was measured by a UV radiometer and was 94.5% and 5.5%, respectively. We previously established a mouse model to study the effect of chronic exposure to SSL on SKH-1 hairless mice [23].

### 2.3. In Vitro Assessment of Sunscreen Components for UV Protection

To measure the efficacy of each sunscreen component for protection against UV irradiation in vitro, we used VITRO-SKIN (IMS Inc., Portland, ME, USA), which effectively mimics the surface properties of human skin. It contains both optimized protein and lipid components and is designed to exhibit a topography, pH, critical surface tension, and ionic strength similar to normal human skin [24,25]. Briefly, VITRO-SKIN membranes were incubated with a liquid mixture of 85% water and 15% glycerin in a closed and humidity-controlled chamber for 20 h prior to sunscreen application. The hydrated VITRO-SKIN membranes were removed from the hydration chamber and placed on a plastic-covered foam block from the IMS VITRO-SKIN Starter Kit (used to simulate the flexibility of the human dermis). Then, 100 µL of lotion alone or lotion with each sunscreen component or combination was rubbed using a gloved finger onto the hydrated VITRO-SKIN membranes. After 1 h, the hydrated VITRO-SKIN membranes were exposed to SSL, which includes both UVA and UVB. The UVA or UVB detector attached to an ILT1700 Research Radiometer (International Light Technologies, Peabody, MA, USA) was placed under the membrane, and then we measured the transmitted energies (W/cm^2^) of UVA and UVB through the membrane. The UVA or UVB energy (W/cm^2^) penetrating the sunscreen-treated groups (Groups 2 to 13) was compared with the vehicle-treated control group 1. The percentage of UV protection (i.e., blockage) was determined as 100-(control group/sunscreen treated group × 100), with the UVA or UVB energy measured as W/cm^2^. The sunscreen-treated groups were divided as follows: Group 1 = SSL with vehicle lotion only; Group 2 = 3% avobenzone (96.65 mM) in lotion with SSL; Group 3 = 10% octocrylene (276.64 mM) in lotion with SSL; Group 4 = 6% oxybenzone (262.88 mM) in lotion with SSL; Group 5 = 7.5% octinoxate (258.26 mM) in lotion with SSL; Group 6 = 5% octisalate (199.74 mM) in lotion with SSL; Group 7 = 12% titanium dioxide (1.502 M) in lotion with SSL; Group 8 = 20% zinc oxide (2.457 M) in lotion with SSL; Group 9 = 10% homosalate (381.16 mM) in lotion with SSL; Group 10 = 3% avobenzone (96.65 mM) + 7.5% octinoxate (258.26 mM) in lotion with SSL; Group 11 = 7.5% octinoxate (258.26 mM) + 5% octisalate (199.74 mM) in lotion with SSL; Group 12 = 7% octocrylene (193.65 mM) + 6.9% zinc oxide (847.87 mM) in lotion with SSL; Group 13 = 3% avobenzone (96.65 mM) + 7% octocrylene (193.65 mM) + 6% titanium dioxide (751.22 mM) in lotion with SSL. All the groups were exposed to SSL.

### 2.4. Animal Care and Maintenance

Female SKH-1 (Crl:SKH1-*Hr^hr^*) hairless mice (strain code 477, Charles River Laboratories, Burlington, MA, USA) were maintained in The Hormel Institute Animal Facilities according to the guidelines approved by the University of Minnesota Institutional Animal Care and Use Committee (IACUC). The IACUC-approved ID is 1712-35398A. Mice were randomly grouped by age and body weight, and 10–15 mice per group (10 mice for groups 1–10 and 15 mice for groups 11–24) were typically used and treated for the criteria achievement of statistical significance without investigator blinding.

### 2.5. The Solar Simulated Light (SSL)-Induced Cutaneous Squamous Cell Carcinoma (cSCC) Mouse Model

SKH-1 hairless mice are unpigmented and immunocompetent. SSL-induced skin carcinogenesis has been extensively studied in SKH-1 hairless mice and is well characterized in dermatologic research. Furthermore, SKH-1 hairless mice allow for the application of topical agents, and the easy visualization of the cutaneous response [26]. SKH-1 hairless mice were exposed to SSL, including UVA and UVB in the critical short wavelength region from 365 nm down to the solar cutoff of 295 nm, with a peak emission at 340 nm. For the topical application of each sunscreen component or combination, we created an oil-in-water emulsion lotion (Patent International Application Number: PCT/US2018/036720; title: skin care formulations and skin cancer treatment) containing each sunscreen component or combination. Eight FDA-approved sunscreen components were examined alone or in combination to confirm their effectiveness in preventing SSL-induced skin cancer. Female SKH-1 hairless mice aged 5–6 weeks (body weight 20–22 g) at the beginning of the study were treated with formulations that included one or more sunscreen component and then exposed to SSL. The lotion vehicle alone or containing one or more sunscreen components was topically applied to the dorsal (i.e., posterior to the base of the neck and anterior to the base of the tail) of the SKH-1 hairless mouse skin 3 times a week prior to 1 h exposure of SSL on the same day. We used a prevention model (Supplementary Figure S2a) where at week 1, the SSL dose was 37 kJ/m^2^ UVA and 1.8 kJ/m^2^ UVB (3 times per week). The dose of SSL was gradually increased at a rate of 10% per week. At week 6, the dose was 60 kJ/m^2^ UVA and 2.9 kJ/m^2^ UVB, and this dose was maintained until week 15. At week 15, the mice were no longer exposed to SSL, but the topical application of lotion with or without the sunscreen component or combination was continued until 29 weeks. The SKH-hairless mice were divided into groups as follows (n = 10 each group; Supplementary Figure S2b): Group 1 = no treatment; Group 2 = vehicle lotion; Group 3 = 3% avobenzone (96.65 mM) in lotion; Group 4 = 10% octocrylene (276.64 mM) in lotion; Group 5 = 6% oxybenzone (262.88 mM) in lotion; Group 6 = 7.5% octinoxate (258.26 mM) in lotion; Group 7 = 5% octisalate (199.74 mM) in lotion; Group 8 = 12% titanium dioxide (1.502 M) in lotion; Group 9 = 20% zinc oxide (2.457 M) in lotion; Group 10 = 10% homosalate (381.16 mM) in lotion. Groups 1–10 were not exposed to SSL whereas Groups 11–24 were exposed to SSL 3× per week. The SSL-treated groups were as follows (n = 15 each group; Supplementary Figure S2c): Group 11 = SSL only; Group 12 = SSL with vehicle lotion only; Group 13 = 3% avobenzone (96.65 mM) in lotion with SSL; Group 14 = 10% octocrylene (276.64 mM) in lotion with SSL; Group 15 = 6% oxybenzone (262.88 mM) in lotion with SSL; Group 16 = 7.5% octinoxate (258.26 mM) in lotion with SSL; Group 17 = 5% octisalate (199.74 mM) in lotion with SSL; Group 18 = 12% titanium dioxide (1.502 M) in lotion with SSL; Group 19 = 20% zinc oxide (2.457 M) in lotion with SSL; Group 20 = 10% homosalate (381.16 mM) in lotion with SSL; Group 21 = 3% avobenzone (96.65 mM) + 7.5% octinoxate (258.26 mM) in lotion with SSL; Group 22 = 7.5% octinoxate (258.26 mM) + 5% octisalate (199.74 mM) in lotion with SSL; Group 23 = 7% octocrylene (193.65 mM) + 6.9% zinc oxide (847.87 mM) in lotion with SSL; Group 24 = 3% avobenzone (96.65 mM) + 7% octocrylene (193.65 mM) + 6% titanium dioxide (751.22 mM) in lotion with SSL. The body weight, tumor volume, and tumor number were measured once a week. The tumor volume (mm^3^) was determined as (length × width^2^) × 0.52, where the length and width were measured in mm as previously described [27]. The average or total tumor volumes were compared between the lotion vehicle-treated mice exposed to SSL and compound in lotion-treated mice exposed to SSL. For example, for avobenzone the average or total tumor volume for the group was 46.1 or 691.5 mm^3^, respectively. The average or total tumor volume for vehicle (lotion) only-treated mice was 169.4 or 2540.5 mm^3^, respectively (Group 12). The percent reduction in tumor volume was 100 × [(169.4 − 46.1)/169.4], or a 72.8% reduction in the average (or total) tumor volume.

### 2.6. Immunohistochemistry (IHC) and Hematoxylin and Eosin (H&E) Staining

After fixation with 4% formaldehyde, skin tissues were embedded in paraffin blocks and subjected to immunohistochemistry (IHC). Briefly, slides from human or mouse skin were baked at 60 °C for 2 h, deparaffinized, and rehydrated. For antigen retrieval, the slides were unmasked by submersion into boiling sodium citrate buffer (10 mM, pH 6.0) for 10 min, and then incubated for 1 h at room temperature. Then, the slides were treated with 3% hydrogen peroxide for 10 min and washed with 1× PBS. The slides were blocked with 10% normal goat serum in 1× PBS in a humidified chamber for 1 h at room temperature and then incubated overnight in a humidified chamber at 4 °C with anti-Ki-67, anti-phospho-TOPK, anti-TOPK, anti-phospho-PRPK, or anti-PRPK as the primary antibody. The slides were washed and hybridized with a biotinylated secondary antibody for 1 h at room temperature. A Vectastain ABC kit (Vector Laboratories, Burlingame, CA, USA) was used to detect the protein targets by following the manufacturer’s instructions. After developing with 3,3′-diaminobenzidine (DAB), the sections were counterstained with hematoxylin. The sections were observed under a LEICA DM IRB microscope and analyzed using the ImagePro Plus software (v.6.1) program (Media Cybernetics, Inc., Rockville, MD, USA). For hematoxylin and eosin (H&E) staining, the slides from human or mouse skin were baked at 60 °C for 2 h, deparaffinized, and rehydrated. The slides were stained with hematoxylin and eosin and then dehydrated and observed under a LEICA DM IRB microscope (200× or 25× magnification).

### 2.7. Immunofluorescence and Confocal Microscopy Analysis

The souse skin tissues were fixed in 4% formaldehyde and then embedded in paraffin blocks before being cut into 4 µm slices and mounted onto slides for analysis. The slides with mouse skin tissues were deparaffinized and hydrated and then permeabilized in 0.5% Triton X-100. The mouse skin tissues were incubated with anti-COX-2, anti-keratin 17, or anti-phospho-EGFR (Tyr1068), and then exposed to an Alexa Fluor 488-labeled secondary antibody. For nuclei staining, the cells were incubated with Fluoro-Gel II with DAPI (4,6-diamidino-2-phenylindole) solution (Electron Microscopy Sciences, Hatfield, PA, USA). The cell images were then analyzed using the Nikon Eclipse TE2000-E confocal microscope equipped with the EZ-C1 software program (Nikon Corporation, Minato-ku, Tokyo, Japan).

### 2.8. Statistical Analyses

The GraphPad Prism 5.0 software (GraphPad Software; La Jolla, CA, USA) was used for all the statistical analyses. All the quantitative results are expressed as mean values ± S.D. Statistically significant differences were obtained using the Student’s test or one-way ANOVA. Values of * *p* < 0.05 or ** *p* < 0.01 were considered to be statistically significant.

## 3. Results

### 3.1. Effectiveness of Sunscreen Components Against Solar Simulated Light (SSL) Irradiation In Vitro

Although sunscreen is widely used, the ability of sunscreens to prevent cSCC remains controversial. Thus, firstly we examined the protective efficacy of each sunscreen component in an in vitro human skin system, VITRO-SKIN (IMS Inc., Portland, ME, USA), as described in Materials and Methods. VITRO-SKIN effectively mimics the surface properties of human skin and is formulated to exhibit a topography, pH, critical surface tension, and ionic strength that is similar to human skin [24,25]. We tested eight FDA-approved sunscreen components (Figure 1a; Supplementary Figure S1) for their ability to block UVA or UVB irradiation. The UV protection% of each sunscreen component in an in vitro human skin system is illustrated in Figure 1b,c. The protective efficacy of each sunscreen component against UVA or UVB rays in vitro is summarized in Table 1. The most effective UV protection against UVA (i.e., >60%) exposure included Groups 2 (71%), 10 (67%), and 13 (81%), compared with the unprotected control group 1. However, no single component or combination was totally protective against UVA exposure. Furthermore, the best protection against UVB (i.e., >60%) included groups 5 (76%), 8 (65%), 10 (68%), 12 (67%), and 13 (81%). Again, no single component or combination of sunscreen components was totally effective against UVB irradiation. However, the protection against UVB was overall greater than the protection against UVA. Notably, only groups 10 and 13 exhibited a greater than 60% protection against both UVA and UVB irradiation. Importantly, the worse protection (i.e., <60%) was observed in Groups 3, 4, 6, 7, 9, and 11. In particular, titanium dioxide is widely used in sunscreens and, in this model, provided only 16% and 49% protection against UVA and UVB, respectively. Additionally, because of serious environmental concerns, Hawaii and certain areas in Florida have banned the use of sunscreen products containing oxybenzone and octinoxate, which are commonly found in many U.S. sunscreen products. In our in vitro system, octinoxate was one of the better components against UVB, which further impacts the number of effective and safe sunscreen ingredients available.

Our results identified the single or combination of FDA-approved sunscreen components that are effective against UVA and UVB irradiation in an in vitro system. We next examined the protective efficacy of single components or a combination of FDA-approved sunscreen components in a chronic SSL-induced skin carcinogenesis study.

### 3.2. Protective Efficacy of Different Candidate Sunscreen Components in a Chronic SSL-Induced Skin Carcinogenesis Study In Vivo

Next, we examined the efficacies of sunscreen components on SSL-induced cSCC development in SKH-1 (Crl:SKH1-*Hr^hr^*) hairless mice. The FDA-approved sunscreen components and concentrations used are presented in Table 2, and the schematic design of the animal study is presented in Supplementary Figure S2a.

**Figure 2:**
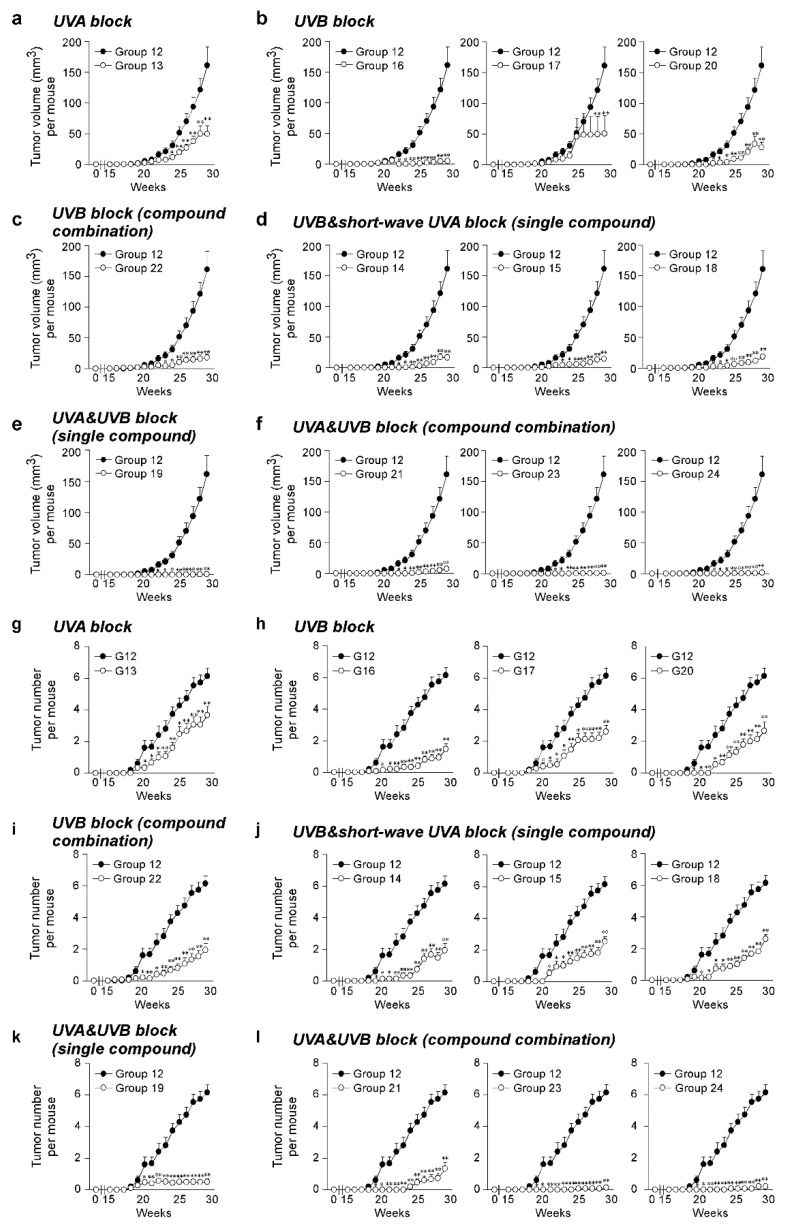
Effectiveness of FDA-approved sunscreen components against cutaneous papilloma and cutaneous squamous cell carcinoma (cSCC) development in vivo. Tumor (**a**–**f**) volume and (**g**–**l**) number per mouse were measured once a week for 29 weeks. Data are shown as means ± S.D. Significant differences were determined by a one-way ANOVA, and asterisks (*^,^ **) indicate a significant (*p* < 0.05 or *p* < 0.01, respectively) difference compared to lotion vehicle group 12, treated with only SSL. SSL-stimulated groups treated with sunscreen components are organized as follows: UVA block ((**a**,**g**) single compound), UVB block ((**b**,**h**) single compound), UVB block ((**c**,**i**) compound combination), UVB and short-wave UVA block ((**d**,**j**) single compound), UVA and UVB block ((**e**,**k**) single compound), and UVA and UVB block ((**f**,**l**) compound combination).

The effect of the SSL exposure on the average tumor volume (Figure 2a–f) and tumor number (Figure 2g–l) over 29 weeks was compared in the groups of mice treated with sunscreen components (Groups 13–24) and a control group of vehicle-treated mice (Group 12). To compare sunscreen components with similar function, the SSL-exposed groups were divided into groups treated with a single UVA blocker, a single UVB blocker, a combination of UVB blockers, a single UVB and short-wave UVA blocker, a single UVA and UVB blocker, and a combination of UVA and UVB blockers. No tumors were observed in any animal not exposed to SSL, regardless of whether untreated, treated with lotion, or treated with each sunscreen component alone in lotion (Groups 1 to 10; Supplementary Figure S2b). The positive controls included mice exposed to SSL without a lotion or sunscreen component (Group 11) and mice exposed to SSL treated with a lotion vehicle (Group 12), and 100% of these mice with or without the topical application of lotion developed tumors (Groups 11 and 12; Supplementary Figure S2c). No statistically significant difference was observed between the positive controls for tumor volume (Supplementary Figure S3a) or tumor number per mouse (Supplementary Figure S3b). Notably, octisalate (Group 17) or titanium dioxide (Group 18) were completely ineffective in protecting against SSL-induced skin cancer (i.e., 100% of mice treated with either compound developed tumors; Supplementary Figure S2c). This finding agrees with the in vitro skin study. Treatment with components containing either a UVA or UVB blocker alone were less protective against SSL-induced skin carcinogenesis, as measured by tumor volume (Figure 2a–d) and tumor number (Figure 2g–j). In contrast, the groups treated with sunscreen components containing both UVA and UVB blockers, including a single compound or a combination of compounds, showed effectiveness in preventing SSL-induced skin carcinogenesis (Figure 2a,f,k–l). In particular, the treatment with zinc oxide alone (Group 19) or in combination with octocrylene (Group 23), or the combination (Group 24) of avobenzone + octocrylene + titanium dioxide had an effectiveness of at least 99% in reducing tumor volume on SSL-induced cSCC development in SKH-1 hairless mice. Whether titanium dioxide played a protective role is questionable based on its ineffectiveness alone (Group 18). The percent reduction in tumor volume was considered as a measure of the effectiveness of the sunscreen component(s) (Table 2). Disturbingly, a high mortality rate of mice was observed in the groups treated with octisalate (Group 17; 66.7% mortality) or octisalate combined with octinoxate (Group 22; 33.3% mortality; Table 2). The mice had to be euthanized early due to severe blistering (Supplementary Figure S2c). Octisalate was relatively ineffective in vitro (45% protection against UVB) and totally ineffective in vivo (100% tumor development).

### 3.3. Effects of Sunscreen Components on Histology and Structure of Skin

**Figure 3:**
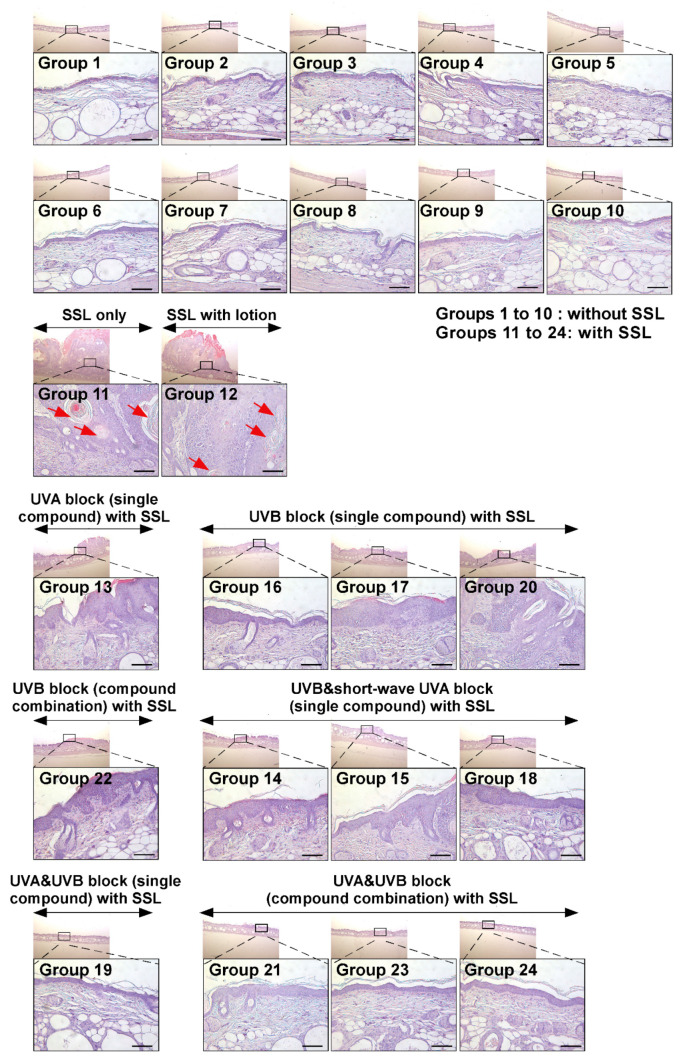
Histological comparison of all the groups of chronic SSL-stimulated SKH-1 hairless mice. At week 29, skin tissues were collected and subjected to H&E staining. Slides were observed under a microscope at 25× or 200× magnification. Scale bars = 100 µm. Red arrows indicate the formation of abundant keratin pearls in cSCC (Groups 11 and 12).

Next, the histological features of mouse skin tissues were compared by H&E staining between mice exposed to SSL with lotion application only (Group 12) and mice exposed to SSL but treated with various sunscreen components (Groups 13 to 24). The histological features of cSCC examined include atypical keratinocytes, dermal invasion, abundant large keratin pearls, and an increased nuclear/cytoplasmic ratio [28]. The results (Figure 3) show that none of the sunscreen components in the absence of SSL caused any damage and or had any effect on skin thickness (Groups 1 to 10; Figure 3; Supplementary Figure S2b). In contrast, chronic SSL exposure induced large epidermal changes and keratinocyte invasion to the dermis (Groups 11 and 12; Figure 3; Supplementary Figure S2c). Additionally, the formation of abundant keratin pearls was observed in the SSL-exposed group (red arrows; Figure 3). Mice in Groups 13, 14, 17, 18, and 22 showed epidermal thickening (Figure 3; Supplementary Figure S2c). These results indicated that sunscreen components blocking only UVA, UVB, or partial UVA and UVB are ineffective in preventing SSL-induced skin damage and carcinogenesis compared with groups treated with sunscreen components that block both UVA and UVB. The most well-protected mice were in Groups 19, 21, 23, and 24, which were treated with sunscreen that blocked both UVA and UVB. Based on these results, blocking both UVA and UVB can prevent SSL-induced epidermal thickening, skin damage, and carcinogenesis.

### 3.4. Effects of Sunscreen Components on Cell Proliferation and Expression of Oncogenic Protein Kinases

**Figure 4:**
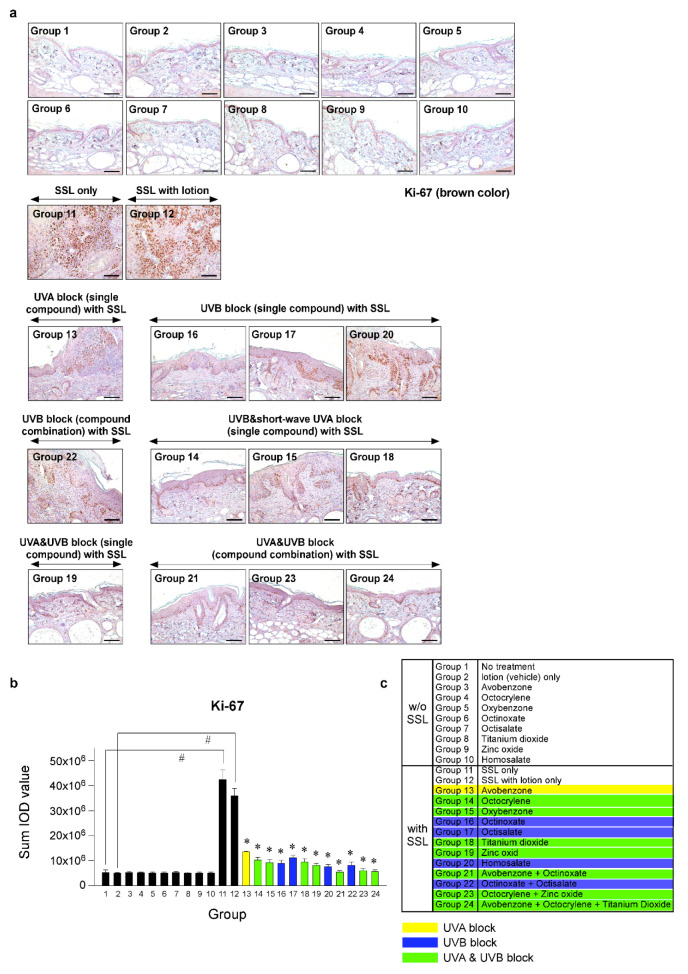
Comparison of the effect of FDA-approved sunscreen components on cell proliferation in SKH-1 hairless mice. (**a**) At week 29, the skin tissues were collected and subjected to immunohistochemistry (IHC) to detect Ki-67 as a proliferation marker. Slides were observed under a microscope at 200× magnification. Scale bars = 100 µm. Brown color indicates proliferating cells. (**b**) The Ki-67 protein levels are presented as the sum of the integrated optical density (IOD) values. The asterisk (^#^) indicates a significant (*p* < 0.01) difference compared to the control group (without SSL). The asterisk (*) indicates a significant (*p* < 0.05) difference compared to the groups treated with the vehicle lotion (with SSL). (**c**) Group information is presented. Groups 1 to 10: without SSL irradiation. Groups 11 to 24: with SSL irradiation.

To investigate the effects of various sunscreen components and combinations on cell proliferation in SSL-stimulated mouse skin, we performed immunohistochemistry (IHC). Photographs comparing tissue samples stained with Ki-67 from mice exposed to SSL and treated or not treated with various sunscreen components and mice not exposed to SSL were compared. Ki-67 staining showed the extent of abnormal cell proliferation, which can lead to skin cancer. The results (Figure 4a–c) indicated that chronic SSL exposure increases the expression of Ki-67 in nuclei of cSCC tissues and results in the proliferation of epidermal cells, such as keratinocytes in Groups 11 and 12, which were exposed to SSL, but not treated with sunscreen (positive controls). The groups treated with single components to block UVA (Group 13) or UVB (Group 20), or a combination to block UVB (Group 22), exhibited only slightly decreased abnormal cell proliferation compared with the groups treated with sunscreen components that blocked both UVA and UVB (Groups 19, 21, 23, 24), which showed the least amount of abnormal proliferation, agreeing with the H&E staining results (Figure 3). The other groups showed moderate increases in abnormal proliferation.

**Figure 5:**
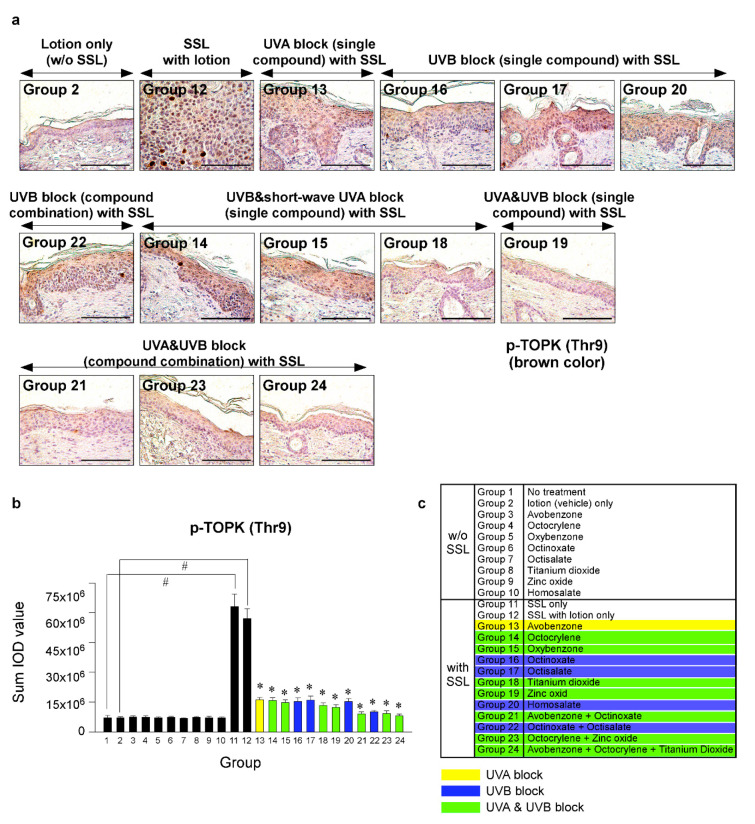
Comparison of the effect of FDA-approved sunscreen components on the phosphorylation of oncogenic kinase T-LAK cell-originated protein kinase (TOPK) in SKH-1 hairless mice. (**a**) At week 29, the skin tissues were collected and subjected to IHC to detect phosphorylated TOPK (Thr9). Slides were observed under a microscope at 400× magnification. Scale bars = 100 µm. Brown color indicates the levels of phosphorylated TOPK (Thr9). (**b**) Phosphorylated TOPK levels are presented as the sum of the IOD values. The asterisk (^#^) indicates a significant (*p* < 0.01) difference compared to the control group (without SSL). The asterisk (*) indicates a significant (*p* < 0.05) difference compared to the groups treated with the vehicle lotion (with SSL). (**c**) Group information is presented. Groups 1 to 10: without SSL irradiation. Groups 11 to 24: with SSL irradiation.

**Figure 6:**
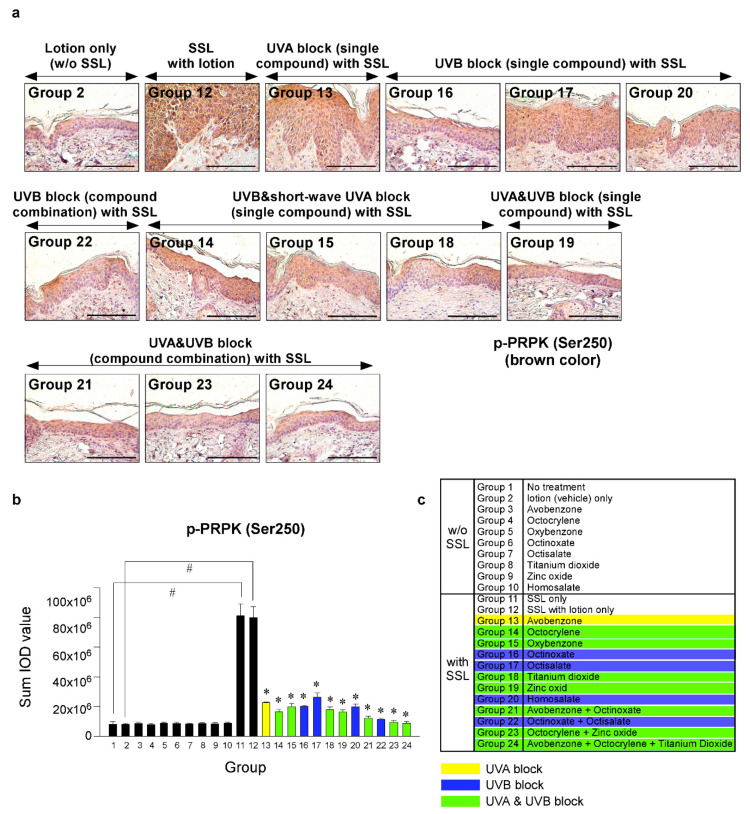
Comparison of the effect of FDA-approved sunscreen components on the phosphorylation of oncogenic kinase p53-related protein kinase (PRPK) in SKH-1 hairless mice. (**a**) At week 29, the skin tissues were collected and subjected to IHC to detect phosphorylated PRPK (Ser250). Slides were observed under a microscope at 400× magnification. Scale bars = 100 µm. Brown color indicates the levels of phosphorylated PRPK. (**b**) Phosphorylated PRPK levels are presented as the sum of the IOD values. The asterisk (^#^) indicates a significant (*p* < 0.01) difference compared to the control group (without SSL). The asterisk (*) indicates a significant (*p* < 0.05) difference compared to the groups treated with the vehicle lotion (with SSL). (**c**) Group information is presented. Groups 1 to 10: without SSL irradiation. Groups 11 to 24: with SSL irradiation.

Recent research results indicated that the levels of phosphorylated TOPK (Thr9) and phosphorylated PRPK (Ser250) are overexpressed in human AKs and SCCs [29]. Furthermore, knocking out the TOPK expression completely blocked the SSL-induced SCC development in SKH-1 hairless mice [29]. Moreover, the results suggested that PRPK is a novel molecular driver associated with the TOPK pathway in skin carcinogenesis. Therefore, we examined the effects of sunscreen components on the expression of TOPK and PRPK, which are associated with malignant progression, in long-term SSL-exposed SKH-1 hairless mice. The results showed that the groups treated with sunscreen components that block both UVA and UVB (Groups 19, 21, 23, 24) exhibited decreased phosphorylated TOPK (Thr9) levels in long-term SSL-stimulated mouse skin (Figure 5a–c). Furthermore, the levels of phosphorylated PRPK (Ser250) were suppressed in SSL-exposed mice treated with sunscreen components or combinations that block both UVA and UVB (Figure 6a–c).

Prostaglandins produced by cyclooxygenase-2 (COX-2) are postulated to be drivers of skin cancer promotion and progression induced by SUV exposure. COX-2 is present in premalignant cutaneous papillomas and cSCCs [30]. Therefore, we examined the effects of sunscreen components on the COX-2 expression. Our immunofluorescence staining results indicated that Groups 14, 15, and 18, which were treated with sunscreen that blocks UVB and short-wave UVA, and also Groups 16 and 22, which were treated with sunscreen that blocks UVB, showed increased levels of COX-2 expression (Supplementary Figure S4a–c). In contrast, Groups 19, 21, 23, and 24 showed baseline levels of COX-2 similar to Group 2 (the lotion-only group without SSL stimulation).

Furthermore, keratin 17 promotes epithelial proliferation and tumor growth in skin, and the downregulation of keratin 17 also attenuates hyperplasia and inflammation [31]. Thus, we investigated the effect of sunscreens on the expression of keratin 17 in the skin. The SSL irradiation significantly increased the expression of keratin 17 in the epidermis of the skin (Supplementary Figure S5a). Importantly, compound combinations (especially groups 23 and 24) that block both UVA and UVB significantly suppressed the expression of keratin 17 in the epidermis of the skin (Supplementary Figure S5a–c).

Tyr 1068 residue in EGFR is a classic autophosphorylation site and subsequently activates MAPK signaling cascades, and the phosphorylation of EGFR at Tyr1068 is associated with the worse grading and prognosis of cSCC [32]. We examined the efficacy of sunscreens on EGFR phosphorylation (Tyr1068). Most sunscreens (Groups 14, 15, 18, 19, 21, 23, and 24) that block both UVA and UVB inhibited the phosphorylated EGFR levels in both the epidermis and dermis areas of the skin (Supplementary Figure S6a). Especially, groups 23 and 24 (compound combination) showed over a 90% inhibitory effect on SSL-increased EGFR phosphorylation in the epidermis and dermis areas of the skin (Supplementary Figure S6a–c).

## 4. Discussion

Solar ultraviolet (SUV) light includes both ultraviolet light A (UVA) and ultraviolet light B (UVB) wavelengths and can induce skin cancers, including cutaneous squamous cell carcinoma (cSCC). Sunscreens are typically used to reduce or block solar UVA and UVB exposure [33]. To our knowledge, the eight most commonly used sunscreen components approved by the FDA have not been critically compared or examined for their effectiveness in preventing skin cancer in a long-term solar simulated light (SSL)-induced mouse model. The components of sunscreens that absorb UV wavelengths probably contribute differentially to the effectiveness of the formulation of a sunscreen, depending on the structure and the concentration of the respective components. Thus, various components of a skin care formulation might provide a different range of protection from SUV-induced skin cancer. The FDA-approved sunscreen components that are commonly used in U.S. products include avobenzone, octocrylene, oxybenzone, octinoxate, octisalate, zinc oxide, titanium dioxide, and homosalate.

Among the FDA-approved eight sunscreen components tested herein, oxybenzone is banned in Hawaii and parts of Florida because of environmental concerns [34]. Furthermore, the use of some sunscreens requires special attention to certain conditions (e.g., swimming pools). For example, chlorine is used as a chemical disinfectant in swimming pools and chlorinated oxybenzone or dioxybenzone caused significantly more cell death compared to unchlorinated controls [35]. Furthermore, retinyl palmitate (RP) is an ester of retinol (vitamin A) and is frequently used in cosmetic products, including sunscreens and anti-aging products. However, recent studies suggested that SKH-1 hairless mice exposed to SSL and administered RP-containing lotions demonstrated an earlier onset of skin tumors and increased incidence and multiplicity of squamous cell skin neoplasms [36]. Moreover, the photo-irradiation of RP with UVA light induces the formation of photodecomposition products, including 5,6-epoxyretinyl palmitate and anhydroretinol. This resulted in DNA damage in a cell-free system and was cytotoxic in cultured mammalian cells [37]. In addition, octinoxate and oxybenzone [38] were found to cause hormonal changes in vivo. However, short-term research in humans did not show any adverse effects. Furthermore, octisalate could cause allergic contact dermatitis [39], and we observed that octisalate treatment increased skin damage and was associated with substantial mouse mortality.

UVB (280–315 nm) irradiation causes direct DNA and RNA damage by inducing covalent bond formation between adjacent pyrimidines, leading to the generation of mutagenic photoproducts, such as cyclopyrimidine dimers and pyrimidine-pyrimidine (6-4) adducts [40,41]. Additionally, UVA (315–400 nm) causes indirect DNA damage through a photo-oxidative stress-mediated mechanism, resulting in the formation of reactive oxygen species, which interact with lipids, proteins, and DNA to generate intermediates that combine with DNA to form harmful adducts [42]. The lack of UVA protection offered by most of the tested sunscreen components is in part due to the limited number of UVA-protective components approved by the FDA. Currently in the U.S., these components include avobenzone, ecamsule, oxybenzone, titanium dioxide, and zinc oxide. Avobenzone has a peak absorption wavelength at 360 nm, but it is inherently photolabile. To overcome the photostability problem, avobenzone can be stabilized with compounds such as octocrylene [43]. Ecamsule has not been approved by the FDA as an active ingredient and can only be used in certain formulations. Thus, better active ingredients with improved UVA protection are needed. In this study, zinc oxide blocked both UVA and UVB and showed an effectiveness against cSCC development (Group 19; Table 2), whereas the commonly used titanium dioxide was totally ineffective. Zinc oxide is not popular because of its white opaque appearance as a physical barrier. To improve the opaque properties, nanoparticles (i.e., average size less than 100 nm) of zinc dioxide are usually used [44]. The use of zinc oxide nanoparticles is still debated because the nanoparticles had phototoxicity effects in HaCaT keratinocytes exposed to UVA irradiation [45,46]. We resolved the opaque properties by decreasing the concentration of zinc oxide and adding other sunscreen components, such as octocrylene in Group 23 and avobenzone and octocrylene in Group 24, producing a sunscreen that blocks UVA and UVB (Table 2).

Human skin is inevitably exposed to repetitive solar irradiation throughout life. Thus, people who are highly sensitive to skin inflammation or actinic keratosis should concentrate on blocking continuous exposure of sunlight, which can cause cutaneous papilloma or cSCC formation. Our results identified sunscreen components that are >99% effective (Groups 19, 23, 24) in preventing SSL-induced skin carcinogenesis in a hairless mouse model.

In conclusion, we created a moisturizing lotion that does not cause or prevent cancer. We then used the lotion as a vehicle to test the effectiveness of eight different sunscreen components alone or in combination in a cSCC mouse model. Our results, including histology and tumor studies, demonstrated that FDA-approved sunscreen components alone or in combination that block both UVA and UVB are the most useful in preventing SSL-induced skin cancer.

## Figures and Tables

**Table 1:** Protective efficacy of sunscreen components in an in vitro human skin system. FDA-approved sunscreens were tested for their protective efficacy against UV irradiation in vitro by using VITRO-SKIN, which effectively mimics the surface properties of human skin as described in Materials and Methods. Data represent mean values ± S.D. of 3 independent experiments. The asterisks (*^,^ **) indicate a significant (*p* < 0.05 or *p* < 0.01, respectively) difference compared to the control group (SSL with lotion only) in UVA measurement. The asterisks (#, ##) indicate a significant (*p* < 0.05 or *p* < 0.01, respectively) difference compared to the control group (SSL with lotion only) in UVB measurement.

Group No.	Component ^†^	Protective Function against UVA and/or UVB	Penetrated UV Energies under the Membrane		% Protection against ^†††^		* *p* < 0.05 or ** *p* < 0.01	^#^*p* < 0.05 or ^##^ *p* < 0.01
			UVA Energy (W/cm^2^) ± SD ^††^	UVB Energy (W/cm^2^) ± SD ^††^	UVA	UVB	UVA	UVB
1	SSL with lotion only	N/A	7.15 × 10^−4^ ± 0.15	0.39 × 10^−4^ ± 0.01	0	0		
2	3% avobenzone with SSL	UVA only	2.10 × 10^−4^ ± 0.10	0.36 × 10^−4^ ± 0.01	71	7	**	
3	10% octocrylene with SSL	UVB/short wave UVA	4.65 × 10^−4^ ± 0.05	0.18 × 10^−4^ ± 0.01	35	55	**	##
4	6% oxybenzone with SSL	UVB/short wave UVA	5.80 × 10^−4^ ± 0.20	0.24 × 10^−4^ ± 0.02	19	40	*	#
5	7.5% octinoxate with SSL	UVB only	7.00 × 10^−4^ ± 0.02	0.09 × 10^−4^ ± 0.01	2	76		##
6	5% octisalate with SSL	UVB only	7.08 × 10^−4^ ± 0.18	0.22 × 10^−4^ ± 0.02	1	45		#
7	12% titanium dioxide with SSL	UVB/short wave UVA	6.00 × 10^−4^ ± 0.02	0.20 × 10^−4^ ± 0.02	16	49	*	##
8	20% zinc oxide with SSL	UVA and UVB	3.10 × 10^−4^ ± 0.20	0.14 × 10^−4^ ± 0.01	57	65	**	##
9	10% homosalate with SSL	UVB only	6.45 × 10^−4^ ± 0.05	0.25 × 10^−4^ ± 0.02	9	37	*	#
10	3% avobenzone + 7.5% octinoxate with SSL	UVA and UVB	2.35 × 10^−4^ ± 0.15	0.13 × 10^−4^ ± 0.01	67	68	**	##
11	7.5% octinoxate + 5% octisalate with SSL	UVB only	5.18 × 10^−4^ ± 0.18	0.18 × 10^−4^ ± 0.01	28	55	*	##
12	7% octocrylene + 6.9% zinc oxide with SSL	UVA and UVB	3.75 × 10^−4^ ± 0.15	0.13 × 10^−4^ ± 0.01	48	67	**	##
13	3% avobenzone + 7% octocrylene + 6% titanium dioxide with SSL	UVA and UVB	1.35 × 10^−4^ ± 0.15	0.07 × 10^−4^ ± 0.01	81	81	**	##

^†^ The lotion alone or lotion with each sunscreen component or combination was rubbed using a gloved finger onto the hydrated VITRO-SKIN membranes. After 1 h, the hydrated VITRO-SKIN membranes were exposed to SSL, which includes both UVA and UVB. The UVA or UVB detector was placed under the membrane, and then we measured the transmitted energies (W/cm^2^) of UVA and UVB through the membrane. ^††^ UVA or UVB energy (W/cm^2^) penetrating the sunscreen-treated groups (Groups 2 to 13) was compared with the vehicle-treated control group 1. ^†††^ The percentage of UV protection (i.e., blockage) was determined as 100 − (control group/sunscreen treated group × 100), with UVA or UVB energy measured as W/cm^2^.

**Table 2:** Summary of tumor volume and number at week 29. Tumor volume and number for each mouse were measured once a week for 29 weeks. The table includes the (1) group identification number, (2) identification of the component used, (3) the percentage of the component used in the vehicle, (4) whether the group was exposed to SSL, (5) the percentage of mice in each group that developed tumors, (6) the total and average volumes of tumors for each group, (7) the total and average number of tumors for each group, (8) the percentage and total number of mice that either died or had to be sacrificed by the end of the study, (9) the calculated percent effectiveness of each sunscreen component or combination. Data are shown as mean values ± S.D. Significant differences were determined by a one-way ANOVA. The asterisk (**) indicates a significant (*p* < 0.01) difference compared to the group treated with only SSL (with lotion vehicle).

Group #	Sunscreen ^†^		SSL ^††^	% of Mice with Tumors	Total/Average Tumor Volume ± SD ^†††^	Total/Average Tumor Number ± SD ^†††^	% Mortality/Number Mice	% Reduction in Tumor Volume ^††††^	Total Mice Number Used/Group	** *p* < 0.01	
	Compound	% Application								Tumor Volume	Tumor Number
1	No treatment	N/A	No	0	0	0	0	N/A	10		
2	Vehicle lotion only	N/A	No	0	0	0	0	N/A	10		
3	Avobenzone	3	No	0	0	0	0	N/A	10		
4	Octocrylene	10	No	0	0	0	0	N/A	10		
5	Oxybenzone	6	No	0	0	0	0	N/A	10		
6	Octinoxate	7.5	No	0	0	0	0	N/A	10		
7	Octisalate	5	No	0	0	0	0	N/A	10		
8	Titanium dioxide	12	No	0	0	0	0	N/A	10		
9	Zinc oxide	20	No	0	0	0	0	N/A	10		
10	Homosalate	10	No	0	0	0	0	N/A	10		
11	SSL only	N/A	Yes	100	2421.1/161.4 ± 31.7	101/6.7 ± 0.6	26.7/4	N/A	15		
12	SSL with lotion only	N/A	Yes	100	2540.5/169.4 ± 30.2	91/6.1 ± 0.5	20/3	N/A	15		
13	Avobenzone	3	Yes	93.3	691.5/46.1 ± 13.4	55/3.7 ± 0.5	6.7/1	72.8	15	**	**
14	Octocrylene	10	Yes	66.7	246.7/16.5 ± 5.3	25/1.7 ± 0.4	0/0	90.3	15	**	**
15	Oxybenzone	6	Yes	86.7	213.7/14.3 ± 3.9	35/2.3 ± 0.3	6.7/1	91.2	15	**	**
16	Octinoxate	7.5	Yes	80	156.0/10.4 ± 1.6	21/1.4 ± 0.4	13.3/2	93.9	15	**	**
17	Octisalate	5	Yes	100	758.4/50.6 ± 29.3	39/2.6 ± 0.4	66.7/10	70.1	15	**	**
18	Titanium dioxide	12	Yes	100	275.6/18.4 ± 3.7	39/2.6 ± 0.3	13.3/2	89.2	15	**	**
19	Zinc oxide	20	Yes	6.7	11.2/0.8 ± 0.7	2/0.13 ± 0.1	0/0	99.6	15	**	**
20	Homosalate	10	Yes	80	415.7/27.7 ± 8.7	39/2.6 ± 0.6	6.7/1	83.6	15	**	**
21	Avobenzone + Octinoxate	3 + 7.5	Yes	53.3	114.3/7.6 ± 2.6	20/1.3 ± 0.4	13.3/2	95.5	15	**	**
22	Octinoxate + Octisalate	7.5 + 5	Yes	80	261.9/17.5 ± 6.0	29/1.9 ± 0.4	33.3/5	89.7	15	**	**
23	Octocrylene + Zinc oxide	7 + 6.9	Yes	13.3	10.3/0.7 ± 0.6	2/0.13 ± 0.09	0/0	99.6	15	**	**
24	Avobenzone + Octocrylene + Titanium Dioxide	3 + 7 + 6	Yes	20	17.9/1.2 ± 0.8	3/0.2 ± 0.1	6.7/1	99.3	15	**	**

^†^ The lotion vehicle alone or containing one or more sunscreen components was topically applied to the dorsal of the SKH-1 hairless mouse skin 3 times a week prior to 1 h exposure of SSL on the same day. ^††^ The mice are treated with SSL at a dose of 37 kJ/m^2^ UVA and 1.8 kJ/m^2^ UVB; this was increased by 10% every week until the dose reached 60 kJ/m^2^ UVA and 2.9 kJ/m^2^ UVB at week 6. The mice are exposed to SSL irradiation for 15 weeks. ^†††^ Tumor volume and tumor number were measured at week 29. Tumor volume (mm^3^) was determined as (length × width^2^) × 0.52. Average or total tumor volumes (or tumor numbers) were compared between the lotion vehicle-treated mice exposed to SSL and the compound in the lotion-treated mice exposed to SSL. ^††††^ The percentage of reduction in the average (or total) tumor volume was determined as 100 × [(control group 12 − sunscreen treated group)/control group 12]. # The SKH-hairless mice were divided into 24 groups. Groups 1 to 10: without SSL irradiation (n = 10 each group). Groups 11 to 24: with SSL irradiation (n = 15 each group).

## Supplementary Materials

The following are available online at https://www.mdpi.com/2073-4409/9/7/1674/s1: Figure S1: Absorbance wavelengths of each FDA-approved sunscreen component; Figure S2: Effectiveness of FDA-approved individual sunscreen components or combinations against SSL-induced skin carcinogenesis in vivo; Figure S3: Tumor volume and number were not different between Group 11 (SSL only) and Group 12 (SSL + lotion vehicle); Figure S4: Effects of sunscreen components on COX-2 expression in epidermis and dermis of mouse skin; Figure S5: Effects of sunscreen components on keratin 17 expression in epidermis and dermis of mouse skin; and Figure S6: Effects of sunscreen components on the expression of phosphorylated EGFR (Tyr1068) in epidermis and dermis of mouse skin; Supplementary Figure Legends.

## Abbreviations

cSCC	cutaneous squamous cell carcinoma
AK	actinic keratosis
SUV	solar ultraviolet
SSL	solar simulated light
