# Supplementary material for: Are FDA-Approved Sunscreen Components Effective in Preventing Solar UV-Induced Skin Cancer?
Source: Cells. 2020 Jul 11;9(7):1674. doi: 10.3390/cells9071674 (PMC7407267; doi:10.3390/cells9071674)
Supplement: Supplementary file 1 [file cells-09-01674-s001.pdf]

<u>**Supplementary Figure Legends**</u>

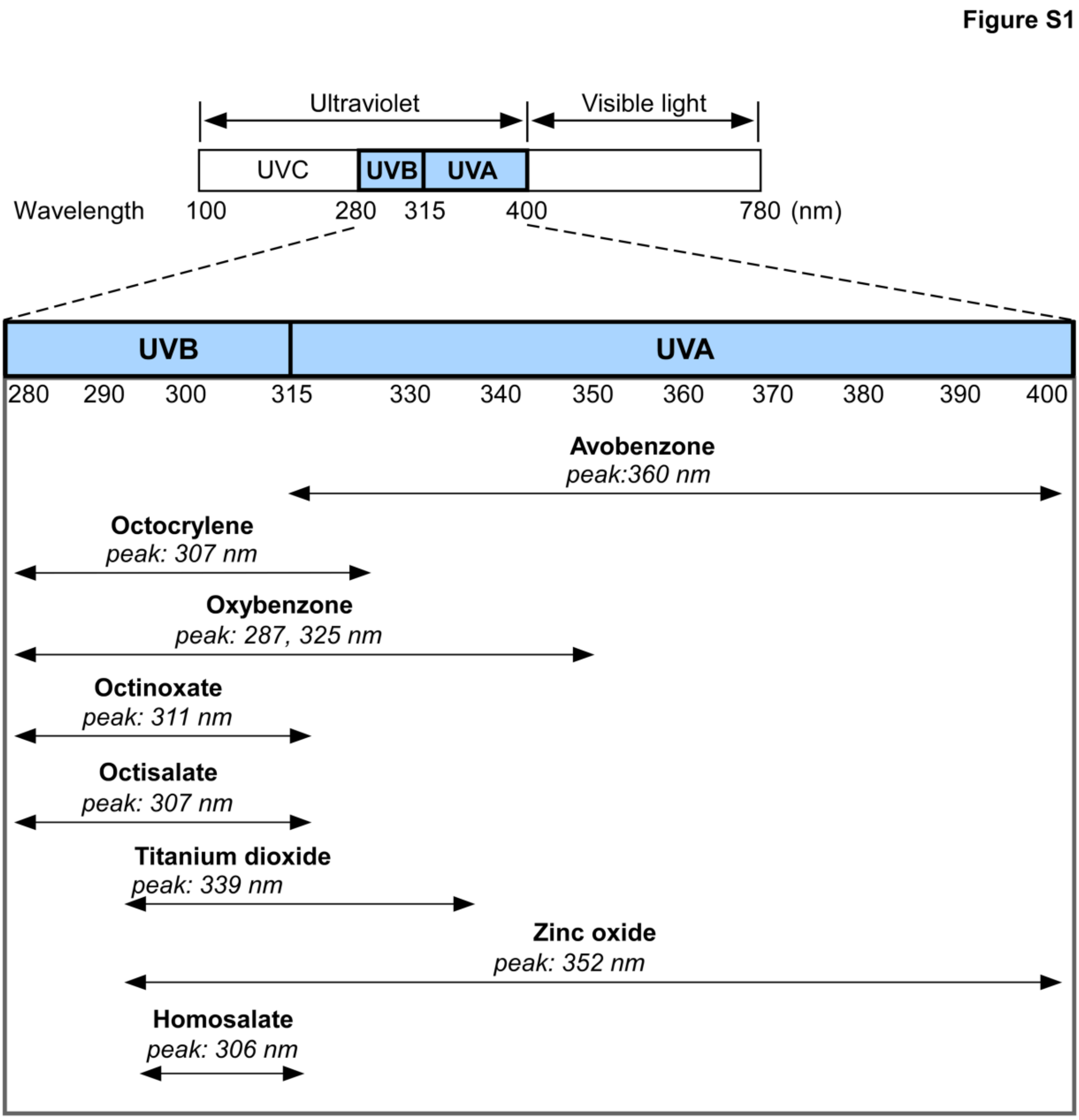

Supplementary Fig. S1. Absorbance wavelengths of each FDA-approved sunscreen component. Maximum peaks of UV absorption are shown.

Figure S2A-C

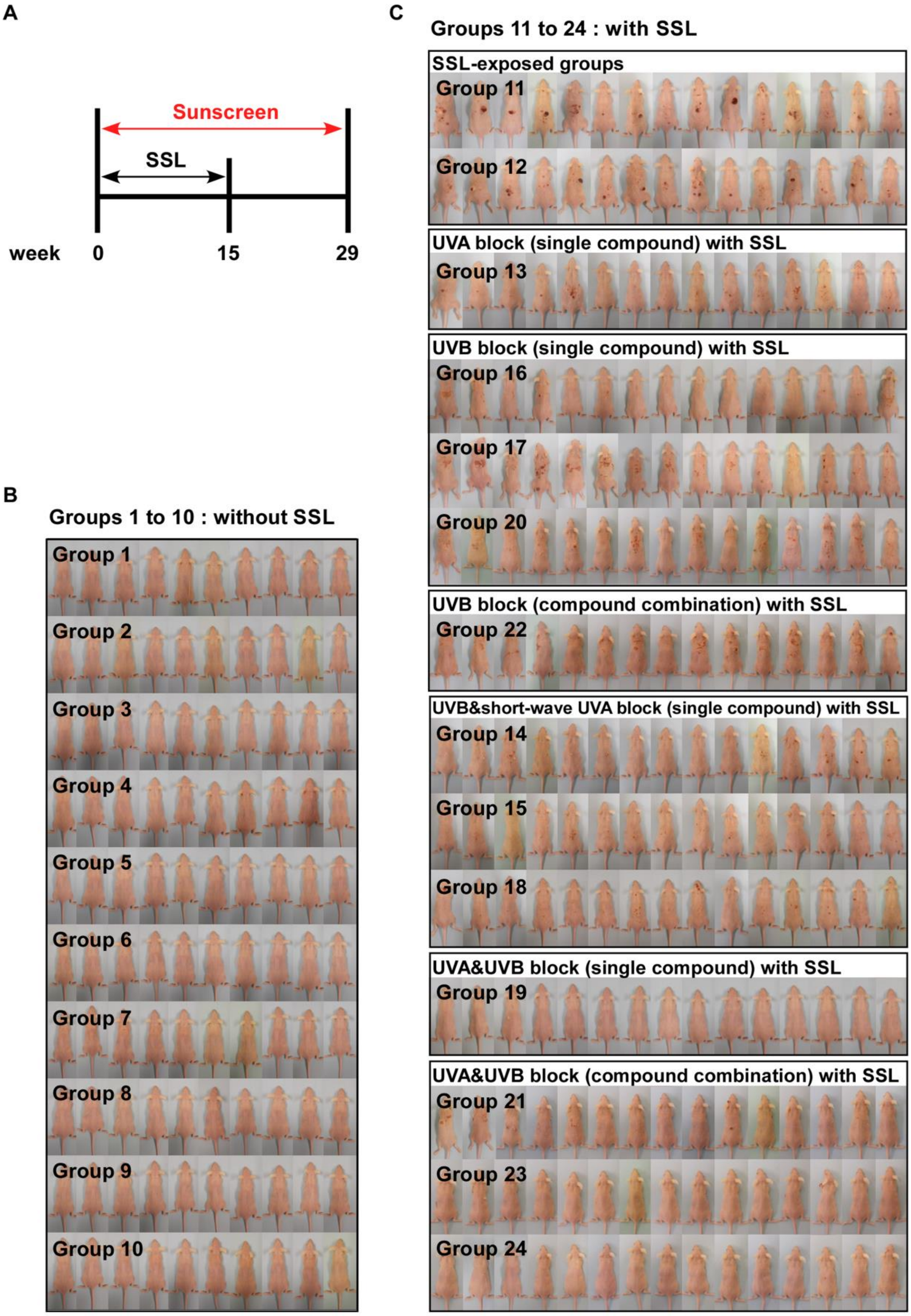

Supplementary Fig. S2. Effectiveness of FDA-approved individual sunscreen components or

combinations against SSL-induced skin carcinogenesis *in vivo*. (**A**) The experimental design for SSL-induced skin carcinogenesis *in vivo*. Lotion alone or containing each sunscreen component or combinations was applied for 29 weeks (3 times a week) to the dorsal area of the SKH-1 hairless mouse skin, posterior to the base of the neck and anterior to the base of the tail before exposure to 1 h of SSL irradiation on the same day. SSL treatment was for 15 weeks and then discontinued. (**B**) Photographs of mice in groups 1 to 10 without SSL exposure. (**c**) Photographs of mice in groups 11 to 24 exposed to SSL.

Figure S3A, B

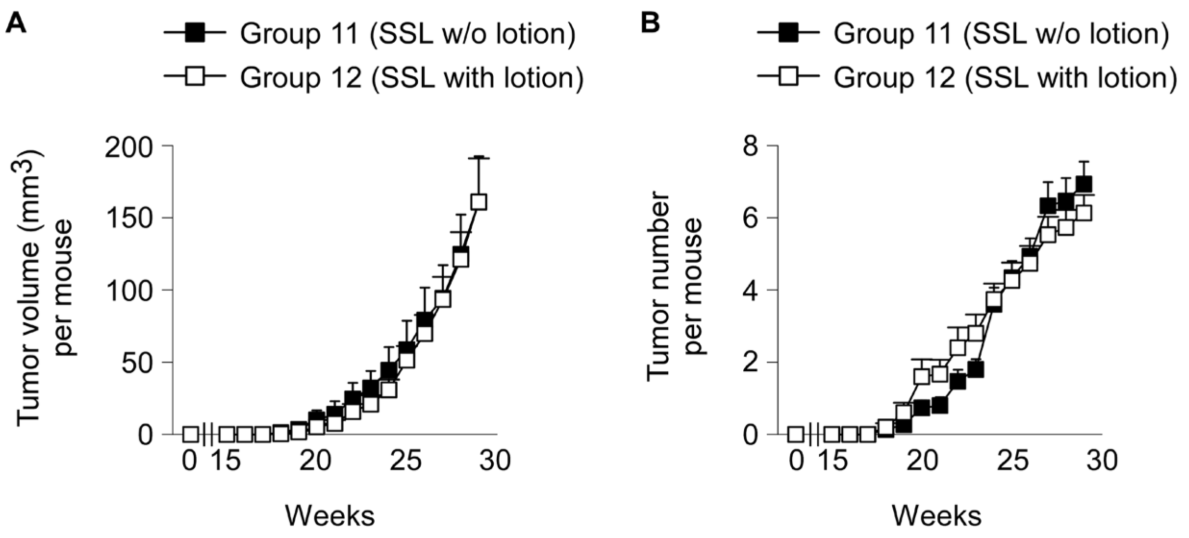

Supplementary Fig. S3. Tumor volume and number were not different between Group 11 (SSL only) and Group 12 (SSL + lotion vehicle). (**A**) Volume and (**B**) number of tumors per mouse were measured once a week for 29 weeks. Data are shown as mean values ± S.D.

Figure S4A-C

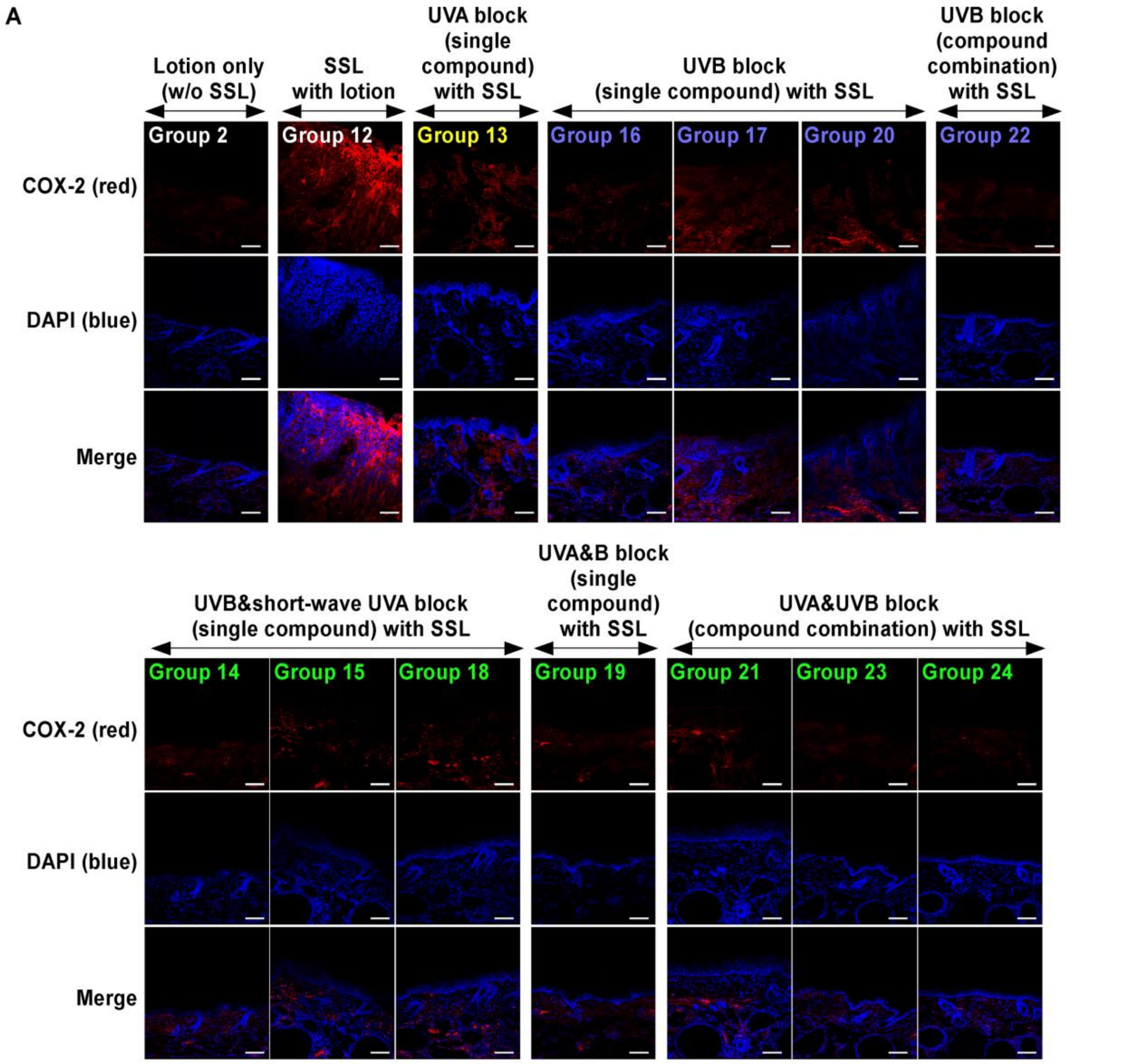

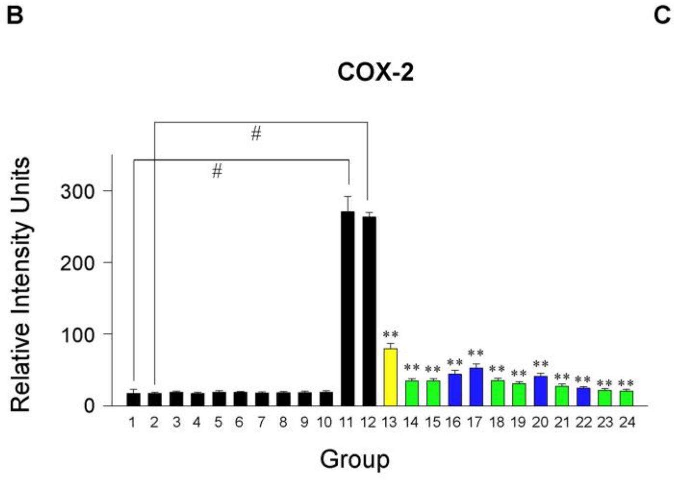

C

w/o SSL	Group 1	No treatment
	Group 2	lotion (vehicle) only
	Group 3	Avobenzone
	Group 4	Octocrylene
	Group 5	Oxybenzone
	Group 6	Octinoxate
	Group 7	Octisalate
	Group 8	Titanium dioxide
	Group 9	Zinc oxide
	Group 10	Homosalate
with SSL	Group 11	SSL only
	Group 12	SSL with lotion only
	Group 13	Avobenzone
	Group 14	Octocrylene
	Group 15	Oxybenzone
	Group 16	Octinoxate
	Group 17	Octisalate
	Group 18	Titanium dioxide
	Group 19	Zinc oxid
	Group 20	Homosalate
	Group 21	Avobenzone + Octinoxate
	Group 22	Octinoxate + Octisalate
	Group 23	Octocrylene + Zinc oxide
	Group 24	Avobenzone + Octocrylene + Titanium Dioxide

UVA block
UVB block
UVA & UVB block

Supplementary Fig. S4. Effects of sunscreen components on COX-2 expression in epidermis

and dermis of mouse skin. (**A**) Mouse skin tissues were fixed in 4% formaldehyde and then immunofluorescence and confocal microscopy analyses were performed as described in Supplementary Materials and Methods. Expression levels of COX-2 are colored in red and nuclei are colored blue. Tissue slides were observed by microscope at 200x magnification. Scale bar = 100 μm. (**B**) The expression levels of COX-2 are presented as the sum of IOD values. The asterisk ($^{\#}$) indicates a significant (p < 0.01) difference compared to the control group (without SSL). The asterisk (**) indicates a significant (p < 0.01) difference compared to the groups treated with the vehicle cream (with SSL). (**C**) Group information is presented. Groups 1 to 10: without SSL irradiation. Groups 11 to 24: with SSL irradiation.

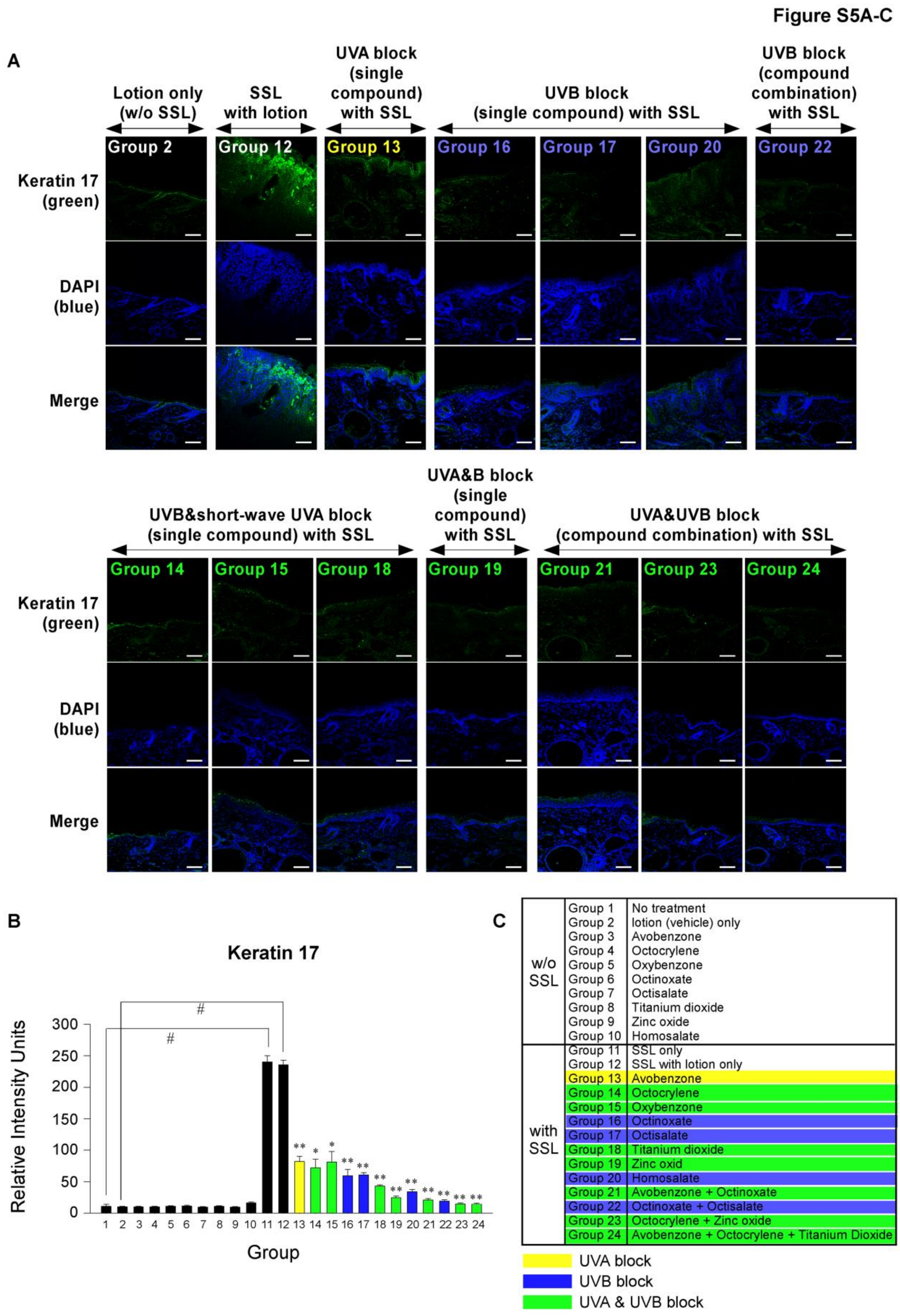

Supplementary Fig. S5. Effects of sunscreen components on keratin 17 expression in

epidermis and dermis of mouse skin. (**A**) Mouse skin tissues were fixed in 4% formaldehyde and then immunofluorescence and confocal microscopy analyses were performed as described in Supplementary Materials and Methods. Expression levels of keratin 17 are colored in green and nuclei are colored blue. Tissue slides were observed by microscope at 200x magnification. Scale bar = 100 μm. (**B**) The expression levels of keratin 17 are presented as the sum of IOD values. The asterisk ($^{\#}$) indicates a significant ($p < 0.01$) difference compared to the control group (without SSL). The asterisks (*, **) indicate a significant ($p < 0.05$ or $p < 0.01$, respectively) difference compared to the groups treated with the vehicle cream (with SSL). (**C**) Group information is presented. Groups 1 to 10: without SSL irradiation. Groups 11 to 24: with SSL irradiation.

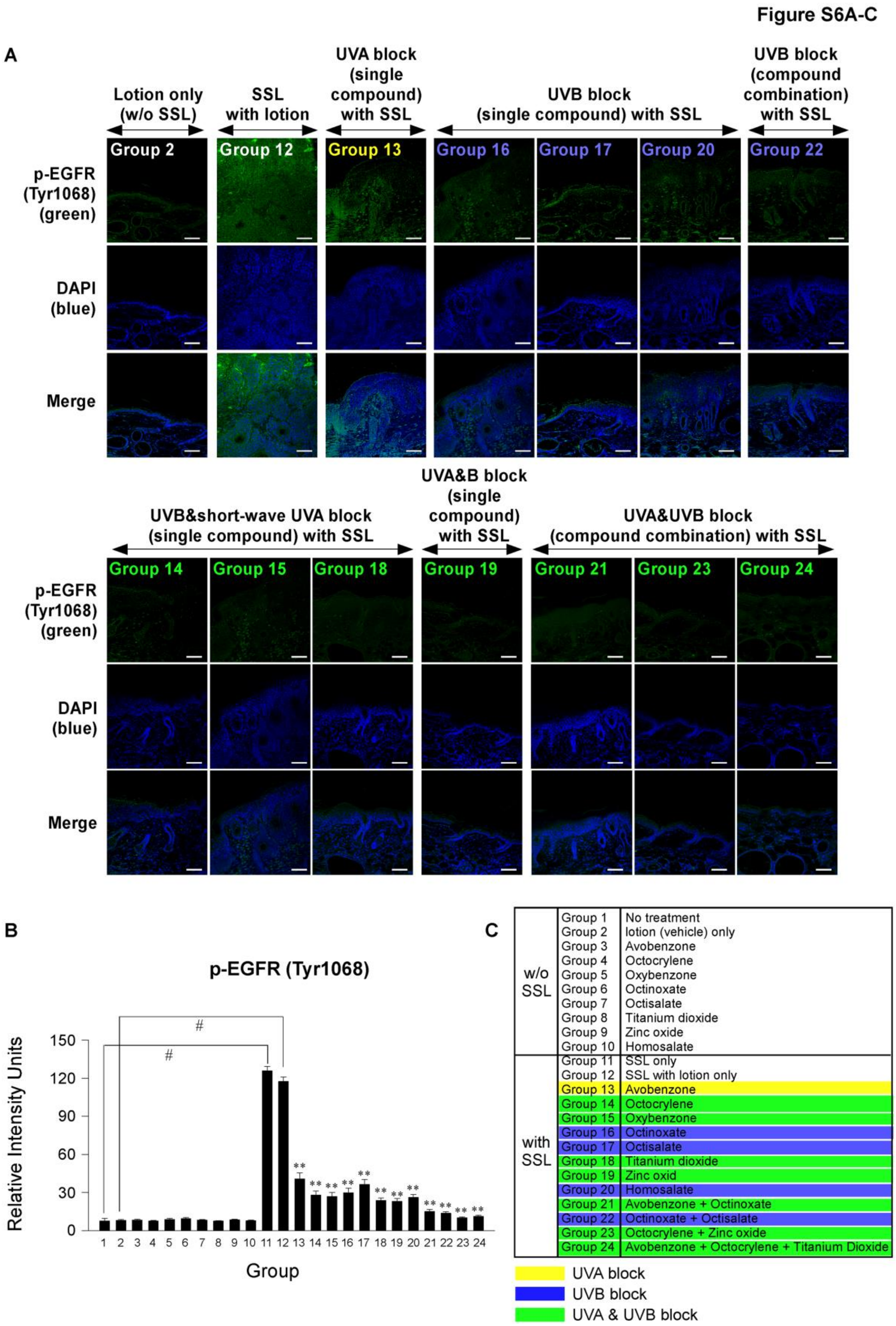

Supplementary Fig. S6. Effects of sunscreen components on the expression of

phosphorylated EGFR (Tyr1068) in epidermis and dermis of mouse skin. (**A**) Mouse skin tissues were fixed in 4% formaldehyde and then immunofluorescence and confocal microscopy analyses were performed as described in Supplementary Materials and Methods. Phospho-EGFR (Tyr1068) levels are colored in green and nuclei are colored blue. Tissue slides were observed by microscope at 200x magnification. Scale bar = 100 μm. (**B**) The phosphorylation levels of EGFR (Try1068) are presented as the sum of IOD values. The asterisk ($^{\#}$) indicates a significant ($p < 0.01$) difference compared to the control group (without SSL). The asterisk (**) indicates a significant ($p < 0.01$) difference compared to the groups treated with the vehicle cream (with SSL). (**C**) Group information is presented. Groups 1 to 10: without SSL irradiation. Groups 11 to 24: with SSL irradiation.
